# Feline gastrointestinal parasitism in Greece: emergent zoonotic species and associated risk factors

**DOI:** 10.1186/s13071-018-2812-x

**Published:** 2018-04-04

**Authors:** Isaia Symeonidou, Athanasios I. Gelasakis, Konstantinos Arsenopoulos, Athanasios Angelou, Frederic Beugnet, Elias Papadopoulos

**Affiliations:** 1 Laboratory of Parasitology and Parasitic Diseases, School of Veterinary Medicine, Faculty of Health Sciences, Aristotle University of Thessaloniki, PO Box: 393, GR 54124 Thessaloniki, Greece; 2 Veterinary Research Institute of Thessaloniki, ELGO-Demeter, GR 57001 Thermi, Thessaloniki Greece; 3 Boehringer Ingelheim, 29 Avenue Tony Garnier, 69007 Lyon, France

**Keywords:** Cats, Gastrointestinal parasites, Greece, Risk factors, Zoonotic potential

## Abstract

**Background:**

Feline gastrointestinal parasitism constitutes an issue of concern for veterinarians since parasites are widespread and affect animals’ health and welfare. Furthermore, some of these pathogens have zoonotic potential. To provide detailed data on the current epizootiology of feline endoparasitism, a multicentric survey was conducted during 2016.

**Methods:**

Faeces from 1150 cats were collected from all regions of Greece and examined by sedimentation and flotation techniques. Possible risk factors including gender, age, ownership status, living conditions and co-infections with other parasites were assessed using binary regression models for each one of the most prevalent parasites.

**Results:**

The overall gastrointestinal parasite prevalence in cats was 50.7%. The study population consisted of cats of both sexes, different age groups, ownership status and living conditions. A total of 10 gastrointestinal parasitic species were detected and up to 5 different parasites were isolated in the same faecal sample. The most frequently identified parasites were *Toxocara cati* (*n* = 278; 24.2%), followed by *Cystoisospora* spp. (*n* = 189; 16.4%), Ancylostomatidae (*n* = 186; 16.2%), *Aelurostrongylus abstrusus* (*n* = 40; 3.5%), *Giardia* spp. (*n* = 26; 2.3%), *Joyeuxiella pasqualei* (*n* = 14; 1.2%), *Capillaria aerophila* (*n* = 8; 0.7%), *Dipylidium caninum* (*n* = 3; 0.2%), *Toxascaris leonina* (*n* = 2; 0.1%) and *Troglostrongylus brevior* (*n* = 2; 0.1%). The occurrence of co-infections was 11.6%. Concerning risk factors, the likelihood of *T. cati* infection was higher for female cats living outdoors and for cats being infected with *Cystoisospora* spp. In the same frame, young, stray, male and free of *A. abstrusus* cats were more likely to be infected with *Cystoisospora* spp. Correspondingly, stray, infected with *Giardia* spp. but free of *Cystoisospora* spp. cats were more likely to be infected with Ancylostomatidae. Regarding *A. abstrusus* infection, a higher probability was reported for cats living outdoors and for cats free of *Cystoisospora* spp., while *Giardia* spp. infections were more common in young and co-infected with Ancylostomatidae animals.

**Conclusions:**

The prevalence of parasitized cats in Greece was high and thus consideration should be paid to control the risk factors, to implement targeted preventive antiparasitic treatments and educate cat owners on the value of prevention for the health and welfare of their cats.

**Electronic supplementary material:**

The online version of this article (10.1186/s13071-018-2812-x) contains supplementary material, which is available to authorized users.

## Background

Cats are common pets and have become part of human families offering companionship [1]. Cats may harbour numerous endoparasites, including protozoa, cestodes, trematodes and nematodes [2]. Among parasites, the gastrointestinal ones may affect cats’ well-being, causing clinical signs like listlessness, dull haircoat, vomiting, diarrhoea, poor growth rate, anaemia and sometimes even death, especially in kittens [3, 4]. Additionally, parasitized cats tend to be more susceptible to viral and bacterial infections, as well as to other diseases, thus their health and welfare status are impaired [5]. Furthermore, some feline parasites, such as *Toxocara cati* and hookworms may cause diseases, such as visceral and ocular *larva migrans* and others, including *Cryptosporidium* spp., *Giardia* spp., *Echinococcus multilocularis* and *Dipylidium caninum* have the potential to infect humans, either *via* direct contact or *via* exposure to contaminated environment [6–9].

Constant monitoring of the active regional prevalence of feline gastrointestinal parasitism and assessing of the specific risk factors involved can provide valuable clues for effective surveillance and prevention [6]. For veterinary practitioners it is important to estimate the changes of their patient to be parasitized in order to implement targeted parasite control schemes. Another aspect of prime importance is the possible public health implication and the appropriate education for applying efficient preventive measures, and thus reducing the zoonotic potential of pet infections [10].

Many studies have investigated the occurrence of feline gastrointestinal parasitism throughout Europe. As reported in two recent multicentric surveys in Europe, these pathogens affect from 30.8% up to 35.1% of owned cats [11, 12]. Depending upon the parasite species, the study population and the diagnostic procedure used, the prevalence estimates vary widely among different countries [12]. It should be emphasized that for stray cat populations the infection rate reaches even up to 100% as a result of inadequate control of parasites and access to intermediate hosts, as indicated by Millan & Casanova [13]. In Greece, there are no comprehensive, large-scale studies regarding the distribution of feline gastrointestinal parasites. All previous studies have been carried out on a small animal population that originated from geographically limited areas of the country, and thus knowledge is fragmented and completely absent for several parts of the country [12, 14–16]. Since Greece is one of the world’s most popular tourist destinations, the continual flow of pets along with the abundant presence of stray cats may have major epidemiological implications.

The objective of this cross-sectional study was to evaluate the current prevalence of feline gastrointestinal parasites in Greece from all regions of the country. Effort was put to assess the emergence of zoonotic cat parasites and to demonstrate their potential interactions. Moreover, this study aimed to designate the risk factors in view of creating a clearer picture of the feline gastrointestinal parasitism in Greece.

## Methods

### Study area and cat population

**Fig. 1:**
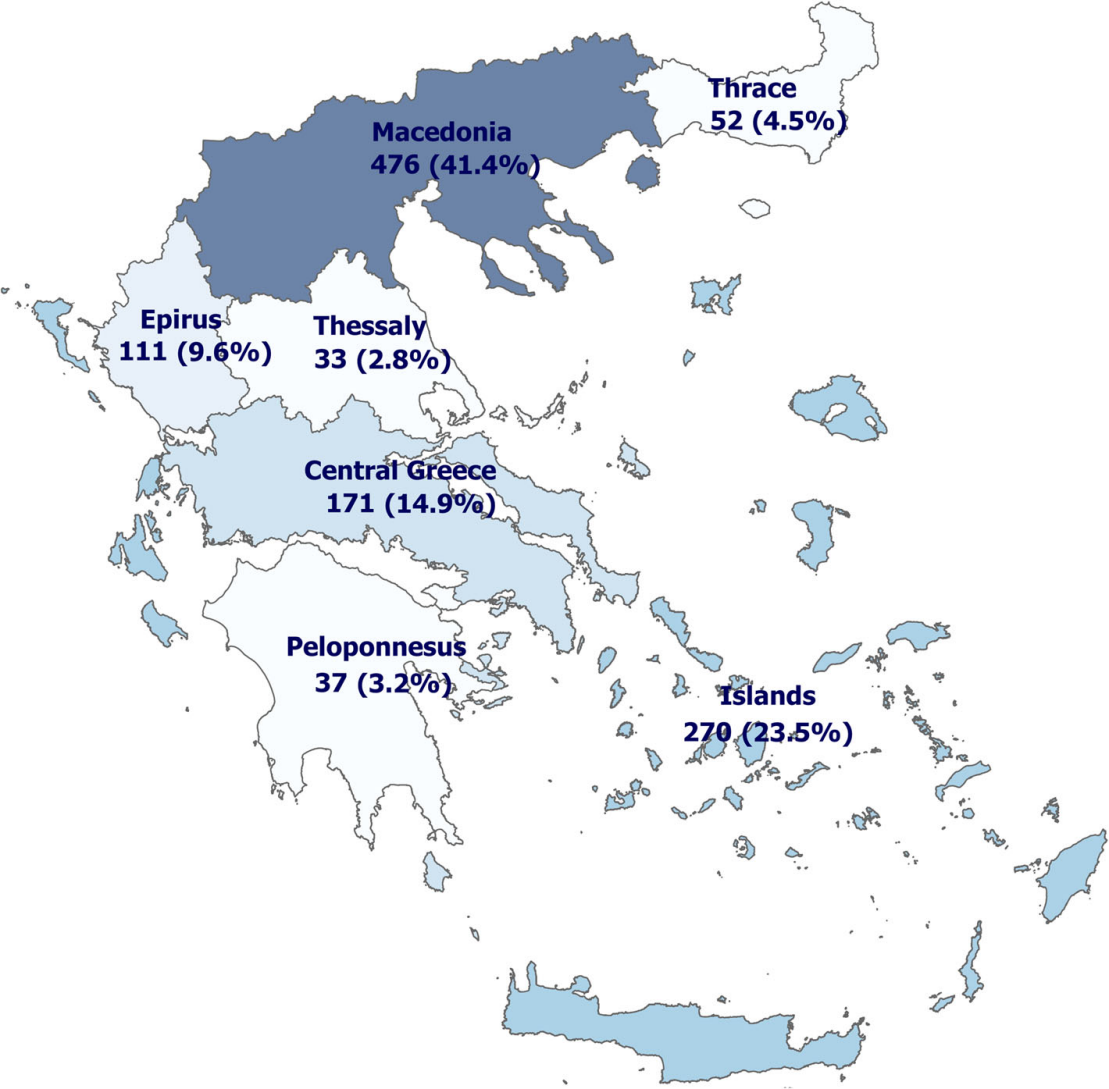
Map displaying the numbers and percentages of feline faecal samples collected per regions of Greece

A total of 1150 faecal samples were collected between January 2016 and November 2016 from clinically healthy cats across Greece, both from the mainland and islands (Fig. 1). Information regarding gender, age, ownership status and living conditions were recorded for each animal sampled. The gender of cats was almost evenly distributed with 553 (48.1%) males and 597 (51.9%) females. The majority of cats were over 6 months old (*n* = 1066 cats; 92.7%), while young animals (< 6 months old) represented 7.3% of the studied population (*n* = 84 cats). Regarding ownership status, samples were collected from owned (*n* = 560; 48.7%) and from stray cats (*n* = 590; 51.3%). Regarding lifestyle, most of the cats were living outdoors (*n* = 926 cats; 80.5%) rather than strictly indoors (*n* = 224 cats; 19.5%). None of the examined animals received any anthelmintic treatment minimum 3 months prior to inclusion.

### Faecal sample collection and coprological methods used

From each individual cat, a faecal sample was collected either at the time of consultation or within 24 h from the cat litter box (when more than one cat per household, animals were isolated in order to accurately identify the sample). Samples were placed individually in plastic containers, labeled with consecutive numbers, stored at 2–6 °C, transferred to the Laboratory of Parasitology and Parasitic Diseases of the School of Veterinary Medicine in Thessaloniki and processed within 48 h.

Initially, each sample was macroscopically examined to detect the possible presence of cestode proglottids and/or adult nematodes. Thereafter, each sample was subjected to a microscopic analysis with a sedimentation and a flotation (ZnSO_4_ 33.2%, specific weight 1.3) technique [17]. Identification of parasites was based on morphological and morphometric features [18, 19]. A cat was considered infected if at least one parasitic element (cyst, oocyst, egg, proglottid or larva) was observed.

### Data handling: statistical analyses

Data were recorded in a Microsoft Excel spreadsheet, appropriately designed for the subsequent analyses (Additional file 1: Table S1, Table S2). Prevalence values were calculated as the proportion of positive animals to the total number of examined animals, whereas the relative prevalence of each parasite species was calculated as the proportion of cats infected with a given parasite species within the total number of positive results. The 95% confidence intervals (CI) of the prevalence values were estimated using the Wilson score interval method. Mean species richness was calculated for gender, age, ownership status and living conditions groups using the Menhinick’s index (D = $\frac{s}{\surd N}$), where *s* is the number of parasite genera and *N* is the total number of cats in the sample.

Initially, five binary logistic regression models were used in order to assess the effects of possible risk factors (gender, age, ownership status, lifestyle, and co-infection with other parasites) and the likelihood that a cat is infected with the most prevalent parasites, namely, *T. cati*, *Cystoisospora* (syn. *Isospora*) spp., Ancylostomatidae (*Ancylostoma/Uncinaria* spp.), *Aelurostrongylus abstrusus* and *Giardia* spp., as described in Model 1:

$$ {\mathrm{Y}}_{\mathrm{P}}=\upalpha +{\upbeta}_1{\mathrm{X}}_1+{\upbeta}_2{\mathrm{X}}_2+{\upbeta}_3{\mathrm{X}}_3+{\upbeta}_4{\mathrm{X}}_4+{\upbeta}_5{\mathrm{X}}_5+{\upbeta}_6{\mathrm{X}}_6+{\upbeta}_7{\mathrm{X}}_7+{\upbeta}_8{\mathrm{X}}_8+{\upbeta}_9{\mathrm{X}}_9\left(\mathrm{Model}\ 1\right) $$

where, Y_P_ is the probability of a cat being infected with the most prevalent parasites (*T. cati*, *Cystoisospora* spp., Ancylostomatidae, *A. abstrusus* and *Giardia* spp.), β_1_ to β_9_, the regression coefficients of gender (X_2_, 0 = male, 1 = female), age (X_3_, 0 = ≤ 6 months, 1 = > 6 months), ownership status (X_4,_ 0 = with owner, 1 = stray), lifestyle (X_1_, 0 = outdoors, 1 = indoors), and *T. cati*, *Cystoisospora* spp., Ancylostomatidae, *A. abstrusus* and *Giardia* spp. infection status (X_5_ to X_9_, respectively, with 0 indicating the absence and 1 the presence of infection).

A stepwise procedure was followed and only predictors that had a significant effect (*P* < 0.05) or tended to have a significant effect (*P* < 0.10) on each individual parasitic infection were used for the final models.

Based on this, the likelihood of (i) *T. cati*, (ii) *Cystoisospora* spp., (iii) Ancylostomatidae, (iv) *A. abstrusus* and (v) *Giardia* spp. infection was estimated using as predictors the regression coefficients of (i) lifestyle, gender and *Cystoisospora* spp. infection status (β_1_, β_2_ and β_6_, respectively), (ii) gender, age, ownership status and *A. abstrusus* infection status (β_2_, β_3_, β_4_ and β_8_, respectively), (iii) ownership status, *Cystoisospora* spp. and *Giardia* spp. infection status (β_4_, β_6_ and β_9_, respectively), (iv) lifestyle and *Cystoisospora* spp. infection status (β_1_, and β_6_, respectively) and (v) age and Ancylostomatidae infection status (β_3_, and β_7_, respectively).

Statistical significance of individual predictors was tested using the Wald *χ*^2^ statistic of their regression coefficients (βs). The Hosmer-Lemeshow (H-L) test, as well as the Cox and Snell *R*^2^ and Nagelkerke *R*^2^ indices were used to assess the goodness-of-fit for each individual model. The ability of the models to correctly predict parasitic infections was assessed by calculating the receiver operating characteristic (ROC) and estimating the areas underneath them (AUC, c-statistic).

The models were validated using an 80–20 cross-validation analysis with the training sample (the sample used to build the model) including 80% of the studied cases and the holdout sample including the remaining 20%; during validation of the model the holdout sample was classified using the coefficients, derived by the model built using the training sample. The classification accuracy of the holdout sample was used to estimate the effectiveness of the model performance for the studied population of cats; a classification accuracy rate within 10% of the training sample was considered to evidence the utility of the model. Moreover, the model was considered efficient when the significance of the predictors’ coefficients in the full-data model matched with the coefficients’ significance in the training sample model.

## Results

### Epizootiology of gastrointestinal parasites in the studied cat population

Overall, 50.7% (583/1150) of the sampled cats were detected to excrete at least one parasitic element. Among examined animals (*n* = 1150 cats), 449 cats (39.0%, 95% CI: 36.3–42.0%) were harbouring only one parasitic genus, while 107 (9.3%, 95% CI: 7.8–11.1%), 25 (2.2%, 95% CI: 1.5–3.2%) and 2 (0.18%, 95% CI: 0–0.01%) cats were found to be infected with species of two, three and more than four different parasite genera, respectively. Overall, eight gastrointestinal and two pulmonary parasite species were detected. *Toxocara cati* was the most prevalent parasite in the studied population (*n* = 278 cats; 24.2%), followed by *Cystoisospora* spp. (*n* = 189 cats; 16.4%), Ancylostomatidae (*n* = 186 cats; 16.2%), *A. abstrusus* (*n* = 40 cats; 3.5%), *Giardia* spp. (*n* = 26 cats; 2.3%), *Joyeuxiella pasqualei* (*n* = 14 cats; 1.2%), *Capillaria aerophila* (syn. *Eucoleus aerophilus*) (*n* = 8 cats; 0.7%), *D. caninum* (*n* = 3 cats; 0.2%), *Toxascaris leonina* and *Troglostrongylus brevior* (*n* = 2 cats each; 0.1%) (Additional file 1: Table S1, Table S2). The prevalence estimates of the detected parasites are summarized in Table 1. Menhinick’s index (D) was 0.54 for young cats and 0.31 for the adult cats. No remarkable differences regarding D were observed between males *vs* females and owned *vs* stray cats (in both cases, 0.38 *vs* 0.41). The range of D values observed in the studied regions was from 0.41 to 0.69. More details, including the number, prevalence (%) of infected (per parasite taxon) cats and the Menhinick’s index in all sampled regions of Greece are shown in Table 2 (Additional file 1: Table S3).

**Table 1:** Prevalence of gastrointestinal and respiratory parasites in the studied cats (*n* = 1150)

Parasite	No. of positive cats	Prevalence (%)	95% CI (%)
*Toxocara cati*	278	24.2	21.8–26.7
*Cystoisospora* spp.	189	16.4	14.4–18.7
Ancylostomatidae	186	16.2	14.2–18.4
*Aelurostrongylus abstrusus*	40	3.5	2.6–4.7
*Giardia* spp.	26	2.3	1.6–3.3
*Joyeuxiella pasqualei*	14	1.2	0.7–2.0
*Capillaria aerophila*	8	0.7	0.4–1.4
*Dipylidium caninum*	3	0.2	0–0.8
*Toxascaris leonina*	2	0.1	0–0.6
*Troglostrongylus brevior*	2	0.1	0–0.6
Total (with at least one parasite)	583	50.7	47.8–53.6

**Table 2:** Number and prevalence (%) of infected (per parasite taxon) cats and mean species richness (Menhinick’s index) in all sampled regions of Greece

Region (no. of examined cats)	*Tc*	*Cystois.* spp.	Ancyl.	*Aa*	*Giar.* spp.	*Jp*	*Ca*	*Dc*	*Tl*	*Tb*	D
Macedonia (*n* = 476)	128 (26.9)	99 (20.8)	12 (2.5)	17 (3.6)	2 (0.4)	8 (1.7)	2 (0.4)	2 (0.4)	1 (0.2)	0 (0)	0.41
Islands (*n* = 270)	68 (25.2)	33 (12.2)	152 (56.3)	11 (4.1)	16 (5.9)	0 (0)	5 (1.9)	0 (0)	0 (0)	1 (0.4)	0.43
Central Greece (*n* = 171)	39 (22.8)	24 (14.0)	14 (8.2)	3 (1.8)	0 (0)	4 (2.3)	0 (0)	0 (0)	1 (0.6)	1 (0.6)	0.54
Epirus (*n* = 111)	24 (21.6)	13 (11.7)	5 (4.5)	6 (5.4)	3 (2.7)	1 (0.9)	1 (0.9)	0 (0)	0 (0)	0 (0)	0.66
Thrace (*n* = 52)	13 (25.0)	5 (9.6)	3 (5.8)	1 (1.9)	0 (0)	1 (1.9)	0 (0.0)	0 (0)	0 (0)	0 (0)	0.69
Peloponnesus (*n* = 37)	5 (13.5)	4 (10.8)	0 (0)	2 (5.4)	0 (0)	0 (0)	0 (0)	0 (0)	0 (0)	0 (0)	0.49
Thessaly (*n* = 33)	1 (3.0)	11 (33.3)	0 (0)	0 (0)	5 (15.2)	0 (0)	0 (0)	0 (0)	0 (0)	0 (0)	0.52

*Abbreviations*: *Tc Toxocara cati*, *Cystois. spp. Cystoisospora* spp., *Ancyl.* Ancylostomatidae, *Aa Aelurostrongylus abstrusus*, *Giar. spp. Giardia* spp., *Jp Joyeuxiella pasqualei*, *Ca Capillaria aerophila*, *Dc Dipylidium caninum*, *Tl Toxascaris leonina*, *Tb Troglostrongylus brevior*, *D* Menhinick’s species richness index

### Effects of risk factors on parasitic infections with *T. cati*, *Cystoisospora* spp., Ancylostomatidae, *A. abstrusus* and *Giardia* spp.

The effects of the studied risk factors forced into the regression models (gender, age, ownership status, living conditions, co-infection with other parasites) and the cross-validation of the models for the five parasite taxa (*Toxocara*, *Cystoisospora*, Ancylostomatidae, *Aelurostrongylus* and *Giardia*) are presented below (Tables 3, 4, 5, 6, 7 and 8).

**Table 3:** Accuracy rate of the full dataset, the training (80%) and the holdout sample (20%) of *Toxocara cati*, *Cystoisospora* spp., Ancylostomatidae, *Aelurostrongylus abstrusus* and *Giardia* spp. models

Observed cases		Predicted cases for the full-data model			Predicted cases for the split-sample validation model					
		Infection status^a^		Percentage correct (%)	Selected cases			Unselected cases		
					Infection status^a^		% correct	Infection status^a^		% correct
		Uninfected	Infected		Uninfected	Infected		Uninfected	Infected	
*Toxocara cati* infection status	Uninfected	874	0	100	705	0	100	169	0	100
	Infected	278	0	0	223	0	0	55	0	0
	Accuracy rate			75.9			76.0			75.4
*Cystoisospora* spp*.* infection status	Uninfected	963	0	100	741	0	100	222	0	100
	Infected	189	0	0	155	0	0	34	0	0
	Accuracy rate			83.6			82.7			86.7
Ancylostomatidae infection status	Uninfected	961	5	99.5	766	2	99.7	197	1	99.5
	Infected	173	13	7.0	134	9	6.3	40	3	7.0
	Accuracy rate			84.5			85.1			83.0
*Aelurostrongylus abstrusus* infection status	Uninfected	1112	0	100	878	0	100	234	0	100
	Infected	40	0	0	31	0	0	9	0	0
	Accuracy rate			96.5			96.6			96.3
*Giardia* spp. infection status	Uninfected	1126	0	100	907	0	100	219	0	100
	Infected	26	0	0	18	0	0	8	0	0
	Accuracy rate			97.7			98.1			96.5

^a^Infection status refers to *Toxocara cati*, *Cystoisospora* spp., Ancylostomatidae, *Aelurostrongylus abstrusus* and *Giardia* spp. predicted infection status in accordance to the observed infection status of the aforementioned parasite species

**Table 4:** Regression coefficients of the predictors used in *Toxocara cati* model for the full dataset (f) and the training sample (ts) used for the validation of the model

	B^a^	SE	Wald	*P*	Odds ratio	95% CI for Exp(B)
Living outdoors (f)	0.98	0.219	19.78	< 0.001	2.651	1.725–4.074
Living outdoors (ts)	0.95	0.244	15.35	< 0.001	2.596	1.611–4.184
Living indoors	Ref*.*					
Male (f)	-0.25	0.140	3.04	0.081	0.783	0.595–1.031
Male (ts)	-0.35	0.157	4.95	0.026	0.705	0.518–0.959
Female	Ref*.*					
No *Cystoisospora* spp. infection (f)	-0.31	0.179	2.95	0.086	0.736	0.519–1.044
No *Cystoisospora* spp. infection (ts)	-0.21	0.202	1.11	0.293	0.808	0.544–1.201
*Cystoisospora* spp. infection	Ref*.*					
Constant (f)	-1.60	0.269	35.48	< 0.001	0.201	
Constant (ts)	-1.62	0.301	29.01	< 0.001	0.197	

*Abbreviations*: *SE* standard error, *CI* confidence interval, *Ref.* reference category

^a^Regression coefficient

**Table 5:** Regression coefficients of the predictors used in *Cystoisospora* spp. model for the full dataset (f) and the training sample (ts) used for the validation of the model

	B^a^	SE	Wald	*P*	Odds ratio	95% CI for Exp(B)	
< 6 months (f)	1.16	0.254	20.97	< 0.001	3.203	1.946–5.272	
< 6 months (ts)	1.27	0.277	20.91	< 0.001	3.546	0.797–1.311	
> 6 months	Ref.						
Owned cats (f)	-0.77	0.170	20.59	< 0.001	0.462	0.331–0.645	
Owned cats (ts)	-0.77	0.189	16.49	< 0.001	0.465	0.321–0.673	
Stray	Ref*.*						
Male (f)	0.33	0.163	4.07	0.044	1.390	1.009–1.913	
Male (ts)	0.13	0.181	0.50	0.479	1.137	0.797–1.620	
Female	Ref.						
No *Aelurostrongylus abstrusus* infection (f)	1.37	0.735	3.47	0.062	3.930	0.931–16.587	
No *Aelurostrongylus abstrusus* infection (ts)	1.15	0.746	2.36	0.125	3.144	0.729–13.560	
*Aelurostrongylus abstrusus* infection	Ref*.*						
Constant (f)	-2.92	0.736	15.72	0.001	0.054		
Constant (ts)	-2.54	0.745	11.66	0.001	0.079		

*Abbreviations*: *SE* standard error, *CI* confidence interval, *Ref.* reference category

^a^Regression coefficient

**Table 6:** Regression coefficients of the predictors used in Ancylostomatidae model for the full data set (f) and the training sample (ts) used for the validation of the model

	B^a^	SE	Wald	*P*	Odds ratio	95% CI for Exp(B)
Owned cats (f)	-2.18	0.231	89.57	< 0.001	0.113	0.072–0.177
Owned cats (ts)	-2.28	0.265	73.79	< 0.001	0.103	0.061–0.172
Stray	Ref.					
No *Giardia* spp. infection (f)	-1.62	0.446	13.16	< 0.001	0.198	0.083–0.475
No *Giardia* spp. infection (ts)	-1.44	0.509	7.97	0.005	0.238	0.088–0.644
*Giardia* spp. infection	Ref.					
No *Cystoisospora* spp. infection (f)	0.59	0.242	5.89	0.015	1.800	1.120–2.894
No *Cystoisospora* spp. infection (ts)	0.68	0.281	5.88	0.015	1.978	1.139–3.433
*Cystoisospora* spp. infection	Ref.					
Constant (f)	0.11	0.483	0.05	0.821	1.115	
Constant (ts)	-0.14	0.555	0.07	0.795	0.866	

*Abbreviations*: *SE* standard error, *CI* confidence interval, *Ref.* reference category

^a^Regression coefficient

**Table 7:** Regression coefficients of the predictors used in *Aelurostrongylus abstrusus* model for the full data set (f) and the training sample (ts) used for the validation of the model

	B^a^	SE	Wald	*P*	Odds ratio	95% CI for Exp(B)
Living outdoors (f)	1.65	0.730	5.07	0.024	5.179	1.238–21.656
Living outdoors (ts)	1.37	0.737	3.47	0.062	3.949	0.932–16.736
Living indoors	Ref.					
No *Cystoisospora* spp. infection (f)	1.46	0.731	3.97	0.046	4.286	1.024–17.946
No *Cystoisospora* spp. infection (ts)	1.17	0.738	2.51	0.113	3.218	0.758–13.662
*Cystoisospora* spp. infection	Ref.					
Constant (f)	-6.10	1.008	36.59	< 0.001	0.002	
Constant (ts)	-5.59	1.011	30.58	< 0.001	0.004	

*Abbreviations*: *SE* standard error, *CI* confidence interval, *Ref.* reference category

^a^Regression coefficient

**Table 8:** Regression coefficients of the predictors used in *Giardia* spp. model for the full data set (f) and the training sample (ts) used for the validation of the model

	B^a^	SE	Wald	*P*	Odds ratio	95% CI for Exp(B)	
< 6 months (f)	1.55	0.612	6.43	0.011	4.721	1.423–15.664	
< 6 months (ts)	1.35	0.680	3.93	0.048	3.844	1.014–14.566	
> 6 months	Ref.						
No Ancylostomatidae infection (f)	-1.98	0.442	20.19	< 0.001	0.138	0.058–0.327	
No Ancylostomatidae infection (ts)	-1.30	0.536	5.89	0.015	0.273	0.095–0.779	
Ancylostomatidae infection	Ref.						
Constant (f)	-2.59	0.288	81.01	< 0.001	0.075		
Constant (ts)	-3.08	0.418	54.26	< 0.001	0.046		

*Abbreviations*: *SE* standard error, *CI* confidence interval, *Ref.* reference category

^a^Regression coefficient

#### *Toxocara cati*

Table 4 presents the risk factors used for the *T. cati* model and their effects on *T. cati* infection status. The likelihood of *T. cati* infection was higher (*B* = 0.98, *df* = 1, *P* < 0.0001) for cats living outdoors in comparison to those living indoors (*c.*2.7 times, 95% CI: 1.7–4.1), although no significant difference was noted between owned and stray cats. Similarly, a 1.3-fold higher (95% CI: 1–1.7) probability for *T. cati* infection was recorded for female cats, which, though, was not statistically significant (*B* = -0.25, *df* = 1, *P* = 0.081). Regarding co-infections, cats infected with *Cystoisospora* spp. were 1.4 times (95% CI: 1–1.9) more likely to be infected with *T. cati* when compared to non-infected ones (*B* = -0.31, *df* = 1, *P* = 0.086). The model provided a good fit to the data with the H-L test being insignificant (*χ*^2^ = 1.85, *df* = 5, *P* = 0.869). Moreover, according to the Omnibus test of coefficients the model had a significant predictive value for *T. cati* infection (*χ*^2^ = 30.509, *df* = 3, *P* < 0.0001) with a c-statistic equal to 0.60 (*P* < 0.001). Cox and Snell *R*^2^-value, and Nagelkerke *R*^2^-value were 0.026 and 0.039, respectively.

#### *Cystoisospora* spp.

Young cats were 3.2 times (95% CI: 1.9–5.3) more likely to be infected with *Cystoisospora* spp. when compared to adult cats (*B* = 1.16, *df* = 1, *P* < 0.0001). Likewise, the probability of *Cystoisospora* spp. infection for stray cats was significantly higher (*B* = -0.77, *df* = 1, *P* < 0.0001) in comparison to owned cats (*c*.2.2 times, 95% CI: 1.6–3.0). In addition, the likelihood of *Cystoisospora* spp. infection was found 1.4 times (95% CI: 1.0–1.9) higher for male cats (*B* = 0.33, *df* = 1, *P* = 0.044). Noticeably, animals free of *Aelurostrongylus abstrusus* were *c.*4 times (95% CI: 0.9–16.6) more likely to be infected with *Cystoisospora* spp. (*B* = 1.37, *df* = 1, *P* = 0.062). Table 5 summarizes the effects of the predictors used in the *Cystoisospora* spp. model. The H-L test was statistically insignificant indicating that the model fits the data well (*χ*^2^ = 0.825, *df* = 5, *P* = 0.975). Moreover, according to the Omnibus test of coefficients, the model was predictive of *Cystoisospora* spp*.* infection (*χ*^2^ = 45.777, *df* = 4, *P* < 0.0001) with a c-statistic equal to 0.65 (*P* < 0.0001). Cox and Snell *R*^2^-value and Nagelkerke *R*^2^-value were 0.039 and 0.066, respectively.

#### Ancylostomatidae

Stray cats were 8.8 times (95% CI: 5.6–13.9) more likely to be infected with Ancylostomatidae when compared to owned cats (*B* = -2.18, *df* = 1, *P* < 0.0001). In the same context, cats infected with *Giardia* spp. were 5.1 times (95% CI: 2.1–12.0) more likely to be parasitized with hookworms when compared to non-infected cats (*B* = -1.62, *df* = 1, *P* = 0.0003). Finally, *Cystoisospora* spp.-free cats were 1.8 times (95% CI: 1.1–2.9) more likely to be infected with Ancylostomatidae (*B* = 0.59, *df* = 1, *P* = 0.015). Table 6 summarizes the effects of the predictors used in the Ancylostomatidae model. The H-L test was statistically insignificant indicating that the model fits the data well (*χ*^2^ = 2.094, *df* = 3, *P* = 0.553). Moreover, according to the Omnibus test of coefficients the model was predictive of *Cystoisospora* spp*.* infection (*χ*^2^ = 146.172, *df* = 3, *P* < 0.0001) with a c-statistic equal to 0.74 (*P* < 0.0001). Cox and Snell *R*^2^-value and Nagelkerke *R*^2^-value were 0.119 and 0.203, respectively.

#### *Aelurostrongylus abstrusus*

Table 7 shows the risk factors assessed in the *A. abstrusus* model. A 5.2 times (95% CI: 1.2–21.7) higher probability of *A. abstrusus* infection was recorded for cats living outdoors when compared to those living indoors (*B* = 1.65, *df* = 1, *P* = 0.024). Analogously, cats free of *Cystoisospora* spp. were 4.3 times (95% CI: 1.0–17.9) more likely to be infected with *A. abstrusus* when compared to infected cats (*B* = 1.46, *df* = 1, *P* = 0.046). The H-L goodness-of-fit test in the *A. abstrusus* model indicated a good fit (*χ*^2^ = 0.045, *df* = 2, *P* = 0.978). A significant predictive value for *A. abstrusus* infection was indicated by the Omnibus test of model coefficients (*χ*^2^ = 13.432, *df* = 2, *P* = 0.001) with ROC analysis showing a c-statistic equal to 0.63 (*P* = 0.006). Cox and Snell *R*^2^-value and Nagelkerke *R*^2^-value were 0.012 and 0.045, respectively.

#### *Giardia* spp.

Table 8 shows the effect size of the risk factors used into *Giardia* spp. model. Young cats were 4.7 times (95% CI: 1.4–15.7) more likely to be infected with *Giardia* spp. when compared to adult cats (*B* = 1.55, *df* = 1, *P* = 0.011). In the same frame, co-infection with Ancylostomatidae was associated with a 7.2 times (95% CI: 3.1–17.2) higher likelihood of *Giardia* spp. infection (*B* = -1.98, *df* = 1, *P* < 0.0001). According to the H-L test, the *Giardia* spp*.* model fitted well the data (*χ*^2^ = 0.000, *df* = 1, *P* = 1.000). Also, the Omnibus test of model coefficients was significant and the model was predictive of *Giardia* spp. infection (*χ*^2^ = 21.547, *df* = 2, *P* < 0.0001) producing a c-statistic equal to 0.72 (*P* = 0.0001). In the same model, Cox and Snell *R*^2^-value and Nagelkerke *R*^2^-value were 0.019 and 0.095, respectively.

## Discussion

Feline gastrointestinal parasitism represents a topic of increasing interest in veterinary medicine. The aim of this extensive survey was to provide a detailed view of the epizootiology of feline gastrointestinal parasites in Greece. To the best of our knowledge, this is the first study in Greece to include such a large sample with a representative geographical distribution of the general cat population, and thus provide prevalence estimates that can be considered reliable and generalizable.

Overall, the prevalence of feline parasitism in Greece with at least one parasite was 50.7% (583/1150 cats). Furthermore, the mean species richness (Menhinick’s index) in the different regions of the country was between 0.41 and 0.69 parasites per cat (Table 2), while a remarkable difference was observed for the mean species richness between young and adult cats. Accordingly, high levels of feline gastrointestinal parasitism have been recorded in recent large-scale multicentric studies conducted in Europe [11, 12]. In the Mediterranean region, studies report similar findings, e.g. 28.0% in Spain [20], 35.0% in Italy [21] and 35.7% in Cyprus [22]. In two small-scale regional surveys in Greece, high levels of feline gastrointestinal endoparasitism have been previously documented, which is in agreement with our findings. Particularly, the occurrence of infection in four islands and Athens was 46.7%, whereas in Crete was 38.1% [14, 16]. The most frequently identified parasites in the studied cat population were *T. cati*, followed by *Cystoisospora* spp., Ancylostomatidae, *A. abstrusus* and *Giardia* spp. These findings are consistent with those reported in several other European countries [21].

Among protozoan parasites, the most commonly identified was *Cystoisospora* spp. (16.4%, *n* = 189 cats). Infections with *Cystoisospora felis* and *Cystoisospora rivolta* were documented as *Cystoisospora* spp., although they can be discriminated using morphological criteria [23]. The study of coccidia in cats in Greece needs further evaluation in the future due to the different pathogenicity of the two species. These parasites may destroy the lining of the intestine and cause mild to severe diarrhoea as well as vomiting and/or decreased appetite [24, 25]*.* Data about *Cystoisospora* spp. prevalence in Europe have been reported for owned cats in many countries, such as Romania (8.9%) [26], northern Germany (7.5%) [27], Portugal (5.0%) [28], Italy (up to 4.5%) [21] and the UK (3.0%) [29], while in stray cats prevalence reaches up to 46.3% of the examined population [30]. The prevalence found in our study (16.4%) was much higher than in previous studies carried out in Greece, i.e. from 6.7 to 9.5% [12, 14, 16]. This is probably due to the inclusion in the present study of a significantly higher number of samples originating from all parts of the country and hence a sharpened view of this infection was demonstrated, contrary to the regional coverage of the other studies. The high prevalence of *Cystoisospora* spp. in the present research implies that coccidia are more likely to pose a threat to cats than previously thought in Greece [12, 14, 16].

Relatively uncommon protozoan parasites found in our study were *Giardia* spp. (2.3%; *n* = 26 cats). Other surveys in Europe employing microscopy have recorded similar prevalences [31]. It should be noted that conventional microscopy is less sensitive for the detection of *Giardia* spp. cysts than other methods, such as immunological and molecular techniques. A meta-analysis demonstrated that the pooled *Giardia* spp. prevalence in feline faeces worldwide is 12.0%, when such techniques are applied [31]. Hence, the actual prevalence of this protozoon may be higher in Greece than the estimated herein. Indeed, an increased prevalence (20.5%) of giardiosis has been recorded in Crete in a study employing an immunofluorescence assay [16]. *Giardia* spp. may induce a range of signs, including diarrhoea, vomiting and anorexia, although many cats remain asymptomatic [29, 32]. It is worth noting that a recent study employing Ion Torrent GM sequencing reported that cats infected with *Giardia* spp. have a different faecal microbiome structure and composition than non-infected ones, suggesting an indirect effect of these protozoans in the metabolism of the upper gastrointestinal tract [33]. It should be highlighted that zoonotic strains have been documented [34], but it is unclear whether close contact with infected cats poses a real risk of infection to owners and shelter staff [32].

The zoonotic protozoan *Toxoplasma gondii* was not detected in the present study [35]. Diagnosis of toxoplasmosis in feline population by microscopy is difficult owing to the short shedding period of this parasite [36]. In addition, its specificity is questionable due to the similar appearance of other Apicomplexa parasites. *Toxoplasma gondii* infections are commonly identified by immunological and molecular techniques, which display higher sensitivity [37]. Serological data regarding feline toxoplasmosis in Greece are scarce and thus the actual presence of this parasitism in Greece is presumably underestimated. Finally, *Tritrichomonas foetus* was not detected in the present study. This protozoan is rarely detected by coprological examination and its presence has been confirmed by molecular methods in Greece with a prevalence of 20.0% [38].

According to our results, *T. cati* was the most prevalent helminth in cats in Greece. The characteristic dark-brown coloured eggs with thick-pitted shells were identified in 24.2% (*n* = 278 cats) of the examined faecal samples [39]. *Toxocara cati* has a direct life-cycle and its eggs can resist adverse climatic conditions and remain infective for years [39, 40]. Moreover, *T. cati* infection arises in kittens due to lactogenic larval transmission [39, 41] while it can also be transmitted through the ingestion of infected paratenic hosts [42]. This particular biology and ecology favours the occurrence of this parasite amongst susceptible hosts and accounts for its cosmopolitan distribution [12, 42]. *Toxocara cati* is one of the most prevalent gastrointestinal parasites of cats globally [11]. Similarly to our study, other authors also point out the high proportion of parasitism by this ubiquitous nematode, especially in kittens [8, 20]. In Europe, the infection rates range between 7.2–83.3% in cats [12, 16, 43]. Accordingly, the high level of infection with *T. cati* (24.0%) was verified in two studies conducted mainly in insular Greece and in Athens [14, 16]. The high prevalence of *T. cati* implies the necessity of effective deworming treatments for animals, particularly given the zoonotic potential of this nematode [10].

*Toxocara cati* eggs are dispersed *via* the animal’s faeces in the environment and mature in soil. When people accidentally ingest the infective eggs, larvae hatch and migrate causing *larva migrans* syndromes, i.e. “visceral *larva migrans*” (VLM) and “ocular *larva migrans*” (OLM), with a wide range of presenting features [40, 42, 44]. It is assumed that *T. cati* may play a greater role in these larval syndromes than appraised and its zoonotic potential should not be underestimated [10, 45]. Studies have indicated that it is associated with both VLM and OLM, particularly in terms of causing permanent liver disorders and ocular lesions [42]. Human toxocarosis has been reported between the most frequently identified zoonoses worldwide [46]. Consequently, the high level of infection with *T. cati* detected in the present study should be considered, as it can have a major public health impact. Moreover, in order to restrain possible zoonotic transmission, the establishment of an informed cat owner population is pivotal [42, 44]. Up-to-date, published data regarding human toxocarosis in Greece is scarce and limited only to some sporadic cases [47–49]. Regarding felines, it is also speculated that *T. cati* infection has an effect on gut microbiota richness and diversity, most likely attributed to the immune-modulatory properties of this ascarid. Indeed, a recent study using cat faeces from Greece, positive and negative for *T. cati* eggs, demonstrated that bacteria belonging to the order *Lactobacillales*, the family *Enterococcaceae* and the genera *Enterococcus* and *Dorea* were more abundant in the infected samples, suggesting that certain differences in the composition of the gut microbiota of *T. cati-*positive *vs T. cati*-negative cats could be present [5].

Other nematodes detected in relatively high percentage were the soil-transmitted Ancylostomatidae (16.2%; *n* = 186). Hookworms usually establish subclinical infections; however, they have an impact on feline health causing retarded growth and failure to thrive. Nevertheless, when heavy parasitism occurs, severe clinical signs, i.e. haemorrhagic enteritis and anaemia, due to the blood-feeding behavior of these nematodes and the resulting ulcerations on the mucosa of the small intestine, manifest [50]. The diverse modes of transmission coupled with the well-documented zoonotic implications of these parasites highlight the importance of our findings [51]. The prevalence of hookworms in the present study was one of the highest recorded among European countries [52]. Although Beugnet et al. [11] recorded 1.4% prevalence for felid hookworms in a multicentric study across Europe, other researchers demonstrated higher infection percentages, e.g. 10.1% in Romania [26], 11.1% in Hungary [53] and, surprisingly, 44.4% in Albania [43]. In Greece, hookworm infections were found with prevalences of up to 6.8% in different regional surveys [14, 16].

Regarding parasitism of the respiratory tract by helminths, *A. abstrusus* was identified in 40 cats (3.5%), while eggs of *C. aerophila* and larvae of *T. brevior* were detected in 8 (0.7%) and 2 (0.1%) cats, respectively. Similarly to our study, *A. abstrusus* was the most frequently diagnosed feline lungworm in Europe, a fact attributed to its broad host range [11, 12]. The estimates of *A. abstrusus* infection may be influenced by the employment of diverse diagnostic methods. It should be indicated that when the Baermann technique is applied, higher prevalence estimates are recorded [52]. Indeed, according to recently conducted research, where the Baermann method was used, markedly higher infection rates reaching 16.7% in Romania [12], 17.4% in Portugal [54], 20.0% in Italy [55, 56], 25.0% in Hungary [12] and 35.8% in Bulgaria [12] have been reported, thus suggesting that these parts of Europe consist of enzootic areas. Accordingly, the occurrence of felid metastrongylids in recent surveys carried out with limited cat populations in Greece was up to 11.0% [12, 15]. Consequently, the actual infection with *A. abstrusus* in Greece is presumably much higher than the one reported in our study. *Capillaria aerophila*, yet with a not entirely clarified biology, has been reported occasionally as a cause of human pulmonary capillariosis [55]. The prevalence of *C. aerophila* observed in this study was lower than the reported prevalence in Italy, which ranges between 1.0–5.0% [21]. As in the case of *A. abstrusus*, the prevalence detected for *Troglostrongylus brevior* in the present study was lower than those reported in other countries of southern Europe [12, 57, 58] and in certain districts of Greece [15], estimated using the Baermann method.

The other ascarid infecting cats, *Toxascaris leonina*, was detected in 0.1% (*n* = 2 cats) of the samples. Our findings are in accordance to other studies from Europe where the prevalence estimate of this nematode was considerably low (0.1–1.7%) [11, 20, 21, 52]. However, in some sporadic studies, contrary to our data, the percentages of infection were 7.2 [53] and 8.0% [14].

Tapeworms belonging to the species *Joyeuxiella pasqualei* (1.2%, *n* = 14 cats) and *D. caninum* (0.2%, *n* = 3 cats) were found at a low prevalence. In Greece, Diakou et al. [14] documented 2.0% prevalence for *D. caninum*. However, there is a considerable intermittent excretion of cestode eggs and proglottids in faeces [2]. These helminths can cause non-specific clinical symptoms from the digestive tract, while the presence of proglottids in the perianal region or in the pet’s environment is aesthetically unpleasant [10, 59].

Recognition of possible risk factors provides valuable clues for the prevention and the implementation of targeted antiparasitic treatments [60]. Primarily, *Cystoisospora* spp. and *Giardia* spp. infections were more common in young animals, being more likely (3.2 and 4.7 times, respectively) to shed oocysts or cysts to the environment than adults [61]. Young animals are more prone to protozoan infections due to an immature immune system. Infected kittens are not able to generate a sufficient immune response and consequently develop more severe clinical signs than older cats [24]. Interestingly, it has been demonstrated that *Cystoisospora* spp. prevalence drops to zero as age progresses, but increases steadily again when the cats are over 17 years-old [62].

In the same frame, Ancylostomatidae prevalence appeared to be significantly higher in stray than in household cats. This result is consistent with other surveys on stray or feral cats in Europe, where the reported prevalence rates of hookworms were 7.2% in Italy [63], 19.1% in Portugal [30] and up to 47.0% in Spain [64]. It is worth noting that a study in Majorca revealed 91.0% prevalence for *Ancylostoma tubaeforme* in feral cats [13], which was attributed to the warm and wet climate of this island, favouring the survival of hookworm larvae [65]. Likewise, infection with *Cystoisospora* spp. was more common in stray cats. Cystoisosporosis is more common in shelters as it has been previously reported [66]. The overall prevalence of these protozoans in stray or feral cats in selected studies conducted in Europe was from 11.8% in Poland [67] and 19.3% in the Netherlands [68] up to 46.3% in Portugal [30]. Due to the parasite's mode of transmission, clinical coccidiosis can be a pervasive problem in shelter environments, presumably due to crowded conditions with poor sanitation and hygiene. Such conditions favour the occurrence of parasitic infections, which are transmitted *via* the faecal oral route [66, 69, 70]. Additionally overcrowding enhances parasitism through immune suppression [71]. Other factors corroborating parasitism in multiple-cat households and catteries are the lack of effective antiparasitic treatment and the fact that young animals are not separated from adult ones [30, 52].

When infected, these free-ranging cats circulate on streets and can contaminate the environment with parasitic elements, thus representing a potential threat to other companion animals and people sharing the same habitat [30]. It is important to note that in Greece, there is a large population of stray cats living in colonies, even in the most visited touristic sites such as islands and archeological places. Furthermore, an increase in pet travel within continental Europe has been recorded due to the removal of border controls under the Schengen Treaty and implementation of the PETS Travel Scheme in the UK. Consequently, dogs and cats travelling to Greece for holidays may be at increased risk of acquiring infections. Cats imported from enzootic regions for specific parasites, e.g. the adoption of a Greek stray, should be promptly visited by a veterinarian and treated with an appropriate anthelmintic [10].

Infection with the cat lungworm, *A. abstrusus*, was strongly associated with animals living outdoors. Regarding *A. abstrusus*, Traversa et al. [72] also found that 38 out of the 40 infected cats had outdoor access, which was estimated as significant after multivariate analysis. In the same frame, Diakou et al. [22] pointed out that all cats infected with *A. abstrusus* in a study in Cyprus had an outdoor lifestyle. This is in accordance with the mode of transmission of this parasite, which utilizes terrestrial gastropods as obligatory intermediate hosts and also a wide range of vertebrates (rodents, lizards, frogs), which in sequence are common preys of cats and serve as paratenic hosts [73]. Likewise, *T. cati* infections were more common in animals living outdoors. In our study, the risk of being *T. cati-*positive was 2.7 times higher for cats with frequent outdoor access compared to those which remained indoors. Similar findings have been demonstrated in other studies [11, 13, 26, 30, 52, 70]. This discrepancy probably reflects differences in feed quality and anthelmintic treatments [13]. Indeed, cats living indoors attract more attention and receive better preventive treatments than the ones living outdoors. Moreover, it may suggest that persistence of *T. cati* eggs in the outdoor environment as well as paratenic hosts play a crucial role in the transmission of this ascarid [18].

Our results confirm concurrent profile of infections in the studied feline population and suggest that the presence of certain parasites increases the infection potential with others. In details, animals infected with Ancylostomatidae were most likely to be infected with *Giardia* spp. and *vice versa*. In the same context, cats free of *Cystoisospora* spp. were at most risk of *A. abstrusus* infection. On the other hand, cats free of *A. abstrusus* were more likely to be infected with *Cystoisospora* spp. Finally, cats infected with *Cystoisospora* spp. were at most risk of being co-infected with *T. cati*. One possible explanation for these outcomes relies to the age, young cats being more infected with coccidia and *Toxocara* than *Aelurostrongylus*. Another explanation could be related to the immune responses. In immunological studies conducted in mice and humans, concerning the interplay of helminths and protozoa [74, 75], it has been proposed that the immune-modulatory abilities of helminths, such as the Th_2_ strong polarization and the provoked generalized immunosuppression, abate the generally antagonistic Th_1_-dependent, cell-mediated immune response against protozoans [76, 77]. Indeed, it has been demonstrated that the strong Th_2_ polarization during a helminth (*Trichinella spiralis*) infection in mice promotes infection due to *Giardia* spp. [78]. This trend of strong immune-modulatory effect on the host has also been attributed to chronic hookworm infections [79]. This interaction implies that certain conditions in the gastrointestinal tract serve as predisposing factors for these parasitoses. Since microbiome, mucosal and systemic immunity are closely linked [80], it is speculated that helminth-associated microbiome further polarizes protozoan infections [33]. Consequently, nematodoses in cats have to be viewed in the context of concurrent protozooses.

Concerning gender, the likelihood of *Cystoisospora* spp. infection was found 1.4 times higher for male cats, while a 1.3 higher probability for *T. cati* infection was recorded for female cats, although not statistically significant (*P* = 0.08). Possible pregnancy in female cats may have an immunosuppressive effect [81], and thus influence gastrointestinal parasite shedding rates. In addition, changes of being parasitized by *T.cati* become greater in females owing their more affiliative behaviour (allorub, allogroom, social sniff and amicable approach) [82].

## Conclusions

In conclusion, this study depicts that gastrointestinal parasites are a common finding in cats in Greece*.* The endoparasitic fauna detected was diversified, which indicates that people in Greece are at risk of exposure to some potentially zoonotic parasitic genera. The presence of felid parasitism depends on the level of awareness among owners, and thus it is recommended to educate cat owners and increase perception of the risks involved. Additionally, the findings reported above emphasize the importance of conducting routine and repeated faecal examinations, even in the well-cared pet population, and administering accurate preventive measures and treatments. Regarding stray cats, it is essential to apply appropriate hygiene in shelters and include antiparasitic control strategies in stray cat neutering campaigns. This study contributes to an improved understanding of the epizootiological patterns of feline parasites in Greece. Future fields of research may comprise the employment of sensitive serological or molecular tools and the further development of prediction models based on the integration of specific risk factors.

## Additional file

Additional file 1: **Table S1.** Information regarding origin, gender, ownership status, living conditions, age and infection status for the various detected parasite taxa for each sampled animal. **Table S2.** Coding for data in Table S1. **Table S3.** Number and prevalence (%) of infected (per parasite taxon) cats in all sampled regions of Greece. (XLSX 78 kb)

## Abbreviations

OLM: ocular *larva migrans*
VLM: viscelar *larva migrans*
